# Crystal structure of the η^4^-ketimine titanium complex (di­phenyl­amido-κ*N*){3-methyl-6-[(4-methyl­phen­yl)(phenyl­aza­nid­yl)methyl­idene]cyclo­hexa-2,4-dien-1-yl-κ^2^ *N*,*C* ^1^}(η^5^-penta­methyl­cyclo­penta­dien­yl)titanium(IV)

**DOI:** 10.1107/S2056989017017455

**Published:** 2018-01-01

**Authors:** Malte Fischer, Marc Schmidtmann, Rüdiger Beckhaus

**Affiliations:** a Institut für Chemie, Fakultät für Mathematik und Naturwissenschaften, Carl von Ossietzky Universität Oldenburg, 26129 Oldenburg, Germany

**Keywords:** crystal structure, titanium, η^4^-ketimine complex, half-sandwich complex

## Abstract

The mol­ecular structure of the title titanium(IV) half-sandwich complex, [Ti(η^5^-C_10_H_15_)(η^4^-C_21_H_19_N)(C_12_H_10_N)], shows a three-legged piano-stool geometry at the central Ti^IV^ atom, comprising of one penta­methyl­cyclo­penta­dienyl ligand, one bidentate ketimine ligand in an η^4^-coordination mode and one monodentate di­phenyl­amide ligand. Except for van der Waals forces, there are no significant inter­molecular inter­actions in the crystal.

## Abstract 2

The mol­ecular structure of the titanium(IV) half-sandwich title complex comprises one penta­methyl­cyclo­penta­dienyl ligand, one di­phenyl­amido ligand and one η^4^-bound ketimine ligand, leading to a three-legged piano-stool geometry.

## Chemical context   

In the course of our recent investigations with respect to the unusual η^4^-coordination mode of the ketimine PhN=C(*p*-tol­yl)_2_ ligand in the coordination sphere of titanium (Fischer *et al.*, 2017 ▸; Loose *et al.*, 2014 ▸), the bonding situation of the ketimine ligand has been of great inter­est. This ligand is bonded with the nitro­gen atom and one of the *ortho*-carbon atoms of one *para*-tolyl moiety to the central titanium(IV) atom, forming five-membered ring structures. Structural details based on the results of X-ray diffraction and of density functional theory calculations at the M06-2X level support the formulation of these complexes as non-classical mono­aza­butadiene complexes. However, the follow-up chemistry with various multiple bond substrates of the complexes with formulae [(η^5^-Cp^#^)Ti(η^4^-C_21_H_19_N)(Cl)] (# = H_5_, Me_5_) shows a hidden η^2^-imine reactivity to five-membered titanacycles (Fischer *et al.*, 2017 ▸), being of high inter­est due to the importance of η^2^-bound imine titanium complexes in industrially relevant hydro­amino­alkyl­ation reaction of alkenes (for a recent review on hydro­amino­alkyl­ation reactions, see: Chong *et al.*, 2014 ▸). In contrast, classical mono­aza­butadiene complexes (Manssen *et al.*, 2017*b* ▸; Scholz *et al.* 1998 ▸, 2004 ▸) show ring-enlargement reactions to seven-membered titanacycles, using similar substrates (Manssen *et al.*, 2017*a* ▸; Scholz *et al.*, 1998 ▸). Moreover, the ligand framework of the non-classical mono­aza­butadiene complexes mentioned above is important for their unexpected reactivities. By derivatization of [(η^5^-Cp*)Ti(η^4^-C_21_H_19_N)(Cl)] with the dialkyl-substituted lithium amide LiN(Me)Cy, the formation of a titanadi­hydro­pyrrole is observed as a result of the 1,3-*H*-shift in the five-membered ring system in addition to the salt metathesis reaction (Fischer *et al.*, 2017 ▸).

Here we report the synthesis and crystal structure of the title compound (η^5^-C_10_H_15_)Ti(η^4^-C_21_H_19_N)(C_12_H_10_N), **1**, synthesized by the reaction of [(η^5^-Cp*)Ti(η^4^-C_21_H_19_N)(Cl)] with the diaryl-substituted lithium amide LiNPh_2_. Compound **1** maintains the η^4^-coordination mode of the ketimine ligand.
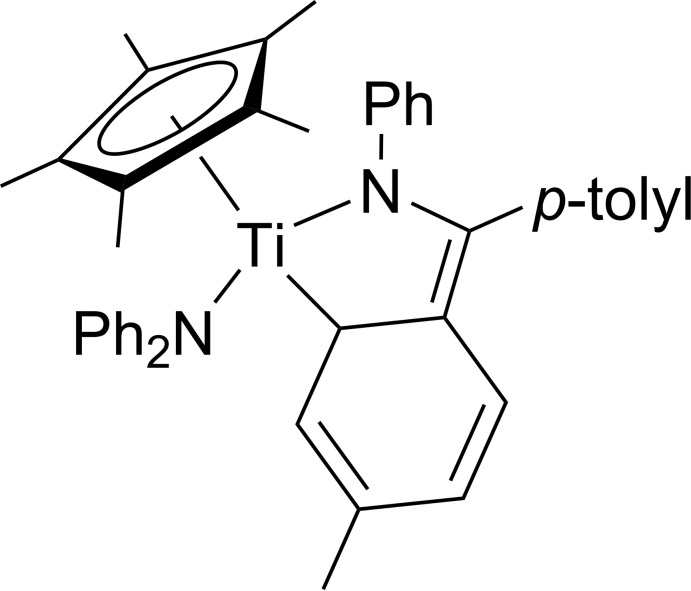


## Structural commentary   

**Figure 1:**
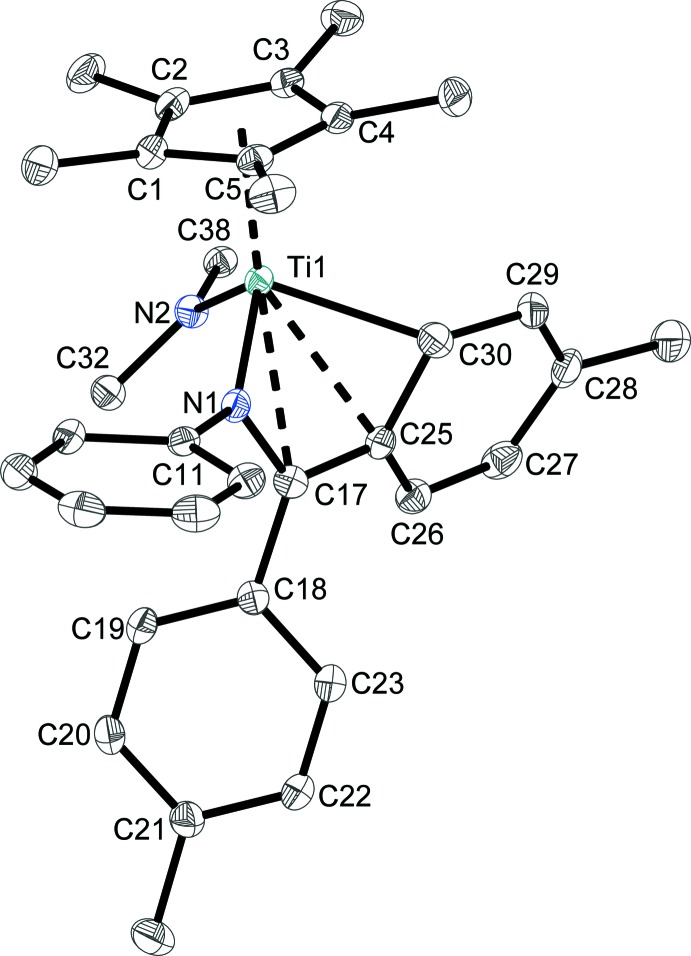
The mol­ecular structure of **1**, with displacement ellipsoids at the 50% probability level. H atoms and phenyl groups of the diphenyl amido moiety have been omitted for clarity.

Fig. 1 ▸ shows the mol­ecular structure of complex **1** for which the η^4^-coordination mode of the ketimine ligand is clearly confirmed. The N1—C17 bond length [1.383 (3) Å] is significantly elongated compared to the free ketimine [1.283 (1) Å; Loose *et al.*, 2014 ▸] and nearly identical to that of the starting complex [(η^5^-Cp*)Ti(η^4^-C_21_H_19_N)(Cl)] [1.393 (2) Å; (Loose *et al.*, 2014 ▸], indicating single-bond character (March, 2007 ▸). The C17—C25 bond length [1.414 (4) Å] is significantly shortened in comparison to the free ketimine [1.497 (1) Å; Loose *et al.*, 2014 ▸]. The sum of angles around C17 {N1—C17—C18 [122.0 (2)°] + N1—C17—C25 [117.0 (2)°] + C18—C17—C25 [120.8 (2)°] = 359.8°} indicates *sp* ^2^-hybridization of this atom. Furthermore, localized C=C double bonds are found in the C25–C30 aromatic ring [C26—C27 = 1.356 (4), C28—C29 = 1.355 (4) Å] in contrast to the well-balanced C—-C distances in the C18–C23 aromatic ring system (≃ 1.39 Å). The central titanium(IV) atom is fourfold coordinated in a considerably distorted tetra­hedral coordination environment, with N1—Ti1—N2 and N1—Ti1—C30 bond angles of 110.42 (9) and 84.23 (9)°, respectively. The Ti1—N1 bond length [1.963 (2) Å] is shorter than the Ti1—N2 bond length [2.009 (2) Å] and indicates weak *p_π_*–*d_π_* electron donor inter­actions. The Ti1—C30 bond length [2.259 (3) Å] as well as the fold angle of the central five-membered ring system (60.6°) are similar to those in other reported mono­aza­butadiene complexes (Manssen *et al.*, 2017*b* ▸; Scholz *et al.*, 1998 ▸, 2004 ▸). The influence of the η^4^-bonding mode of the ketimine ligand can be analysed by the difference Δ = [(Ti1—C17 + Ti1—C25)/2 – (Ti1—N1 + Ti1—C30)/2] = 0.386 Å (Scholz *et al.*, 1998 ▸). This value is in good agreement with the starting material (0.326 Å; Loose *et al.*, 2014 ▸) and other related complexes. The terms *prone* and *supine* are employed to describe the mode of the monoazadiene orientation in the envelope structure of **1**, as summarized by Nakamura *et al*. (2001 ▸). Generally, for mono­aza­butadiene complexes *prone* and *supine* isomers are known. The mol­ecular structure of **1** shows the *supine* isomer.
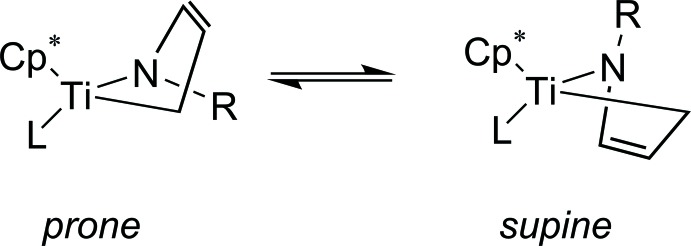


## Supra­molecular features   

**Figure 2:**
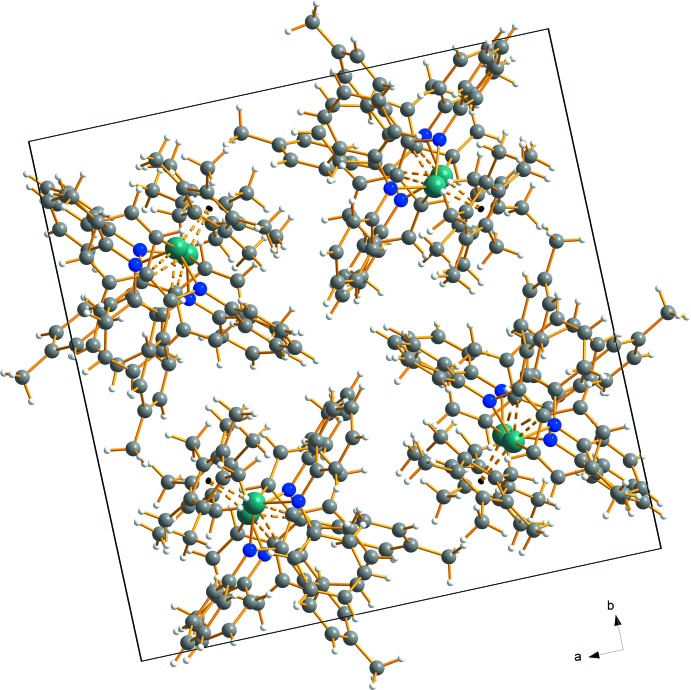
A view along the *c* axis, showing the packing of the mol­ecules in the crystal structure of complex **1**. No significant supra­molecular features can be observed. Colour code: C grey, H colourless, N blue and Ti turquoise spheres.

There are no significant supra­molecular features in the crystal structure of **1.** The crystal packing, shown in Fig. 2 ▸, appears to be dominated by van der Waals inter­actions only.

## Synthesis and crystallization   

All operations were carried out under a dry nitro­gen atmos­phere using Schlenk techniques or in a glove box. The η^4^-ketimine complex [(η^5^-Cp*)Ti(η^4^-C_21_H_19_N)(Cl)] and lithium diphenyl amide were prepared according to published procedures (Fischer *et al.*, 2017 ▸; Hatakeyama *et al.*, 2012 ▸). Solvents were dried according to standard procedures over Na/K alloy with benzo­phenone as indicator and distilled under a nitro­gen atmosphere.

[(η^5^-Cp*)Ti(η^4^-C_21_H_19_N)(Cl)] (0.500 g, 0.992 mmol) and lithium diphenyl amide (0.174 g, 0.992 mmol) were dissolved in 12 ml of tetra­hydro­furan. After stirring the reaction mixture for 16 h at room temperature, the solvent was evaporated in a vacuum. The residue was dissolved in 12 ml of toluene, filtered, and the precipitate of LiCl was washed with toluene (2 ×10 ml). The combined filtrates were evaporated in a vacuum and the residue was recrystallized from *n-*hexane to yield complex **1** as dark-red prisms in 15% crystalline yield.

## Refinement   

Crystal data, data collection and structure refinement details are summarized in Table 1 ▸. Hydrogen atoms bonded to carbon atoms, with the exception of H30 bonded to the *ortho-*carbon atom that is bonded to titanium, were located from difference-Fourier maps but were subsequently fixed in idealized positions using appropriate riding models. Atom H30 was refined freely. The absolute structure was determined (Parsons *et al.*, 2013 ▸) by using 3640 quotients.

## Supplementary Material

Crystal structure: contains datablock(s) I. DOI: 10.1107/S2056989017017455/wm5424sup1.cif

Structure factors: contains datablock(s) I. DOI: 10.1107/S2056989017017455/wm5424Isup2.hkl

CCDC reference: 1589353

Additional supporting information:  crystallographic information; 3D view; checkCIF report

## Figures and Tables

**Table 1:** Experimental details

Crystal data	
Chemical formula	[Ti(C_10_H_15_)(C_21_H_19_N)(C_12_H_10_N)]
*M*_r_	636.70
Crystal system, space group	Tetragonal, *P* 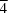 2_1_ *c*
Temperature (K)	100
*a*, *c* (Å)	20.0633 (4), 16.8156 (4)
*V* (Å^3^)	6768.9 (3)
*Z*	8
Radiation type	Mo *K*α
μ (mm^−1^)	0.29
Crystal size (mm)	0.40 × 0.14 × 0.14

Data collection	
Diffractometer	Bruker APEXII CCD
Absorption correction	Multi-scan (*SADABS*; Krause *et al.*, 2015 ▸)
*T*_min_, *T*_max_	0.832, 1.000
No. of measured, independent and observed [*I* > 2σ(*I*)] reflections	152032, 9906, 8703
*R*_int_	0.093
(sin θ/λ)_max_ (Å^−1^)	0.704

Refinement	
*R*[*F* ^2^ > 2σ(*F* ^2^)], *wR*(*F* ^2^), *S*	0.043, 0.110, 1.07
No. of reflections	9906
No. of parameters	426
H-atom treatment	H atoms treated by a mixture of independent and constrained refinement
Δρ_max_, Δρ_min_ (e Å^−3^)	0.65, −0.53
Absolute structure	Flack *x* determined using 3640 quotients [(*I* ^+^)−(*I* ^−^)]/[(*I* ^+^)+(*I* ^−^)] (Parsons *et al.*, 2013 ▸)
Absolute structure parameter	0.003 (8)

Computer programs: *APEX2* and *SAINT* (Bruker, 2015 ▸), *SHELXT* (Sheldrick, 2015*a* ▸), *SHELXL2014* (Sheldrick, 2015*b* ▸), *DIAMOND* (Brandenburg & Putz, 2006 ▸) and *publCIF* (Westrip, 2010 ▸).

## supplementary crystallographic information

### Crystal data

**Table:**

[Ti(C_10_H_15_)(C_21_H_19_N)(C_12_H_10_N)]	*D*_x_ = 1.250 Mg m^−^^3^
*M**_r_* = 636.70	Mo *K*α radiation, λ = 0.71073 Å
Tetragonal, *P*42_1_*c*	Cell parameters from 9899 reflections
*a* = 20.0633 (4) Å	θ = 2.3–27.7°
*c* = 16.8156 (4) Å	µ = 0.29 mm^−^^1^
*V* = 6768.9 (3) Å^3^	*T* = 100 K
*Z* = 8	Tetragonal prism, dark red
*F*(000) = 2704	0.40 × 0.14 × 0.14 mm

### Data collection

**Table:**

Bruker APEXII CCD diffractometer	8703 reflections with *I* > 2σ(*I*)
Radiation source: sealed tube	*R*_int_ = 0.093
φ and ω scans	θ_max_ = 30.0°, θ_min_ = 1.4°
Absorption correction: multi-scan (*SADABS*; Krause *et al.*, 2015)	*h* = −28→28
*T*_min_ = 0.832, *T*_max_ = 1.000	*k* = −28→28
152032 measured reflections	*l* = −23→23
9906 independent reflections	

### Refinement

**Table:**

Refinement on *F*^2^	Secondary atom site location: difference Fourier map
Least-squares matrix: full	Hydrogen site location: difference Fourier map
*R*[*F*^2^ > 2σ(*F*^2^)] = 0.043	H atoms treated by a mixture of independent and constrained refinement
*wR*(*F*^2^) = 0.110	*w* = 1/[σ^2^(*F*_o_^2^) + (0.060*P*)^2^ + 2.*P*] where *P* = (*F*_o_^2^ + 2*F*_c_^2^)/3
*S* = 1.07	(Δ/σ)_max_ = 0.001
9906 reflections	Δρ_max_ = 0.65 e Å^−^^3^
426 parameters	Δρ_min_ = −0.53 e Å^−^^3^
0 restraints	Absolute structure: Flack *x* determined using 3640 quotients [(*I*^+^)-(*I*^-^)]/[(*I*^+^)+(*I*^-^)] (Parsons *et al.*, 2013)
Primary atom site location: structure-invariant direct methods	Absolute structure parameter: 0.003 (8)

### Special details

**Table:**

Geometry. All esds (except the esd in the dihedral angle between two l.s. planes) are estimated using the full covariance matrix. The cell esds are taken into account individually in the estimation of esds in distances, angles and torsion angles; correlations between esds in cell parameters are only used when they are defined by crystal symmetry. An approximate (isotropic) treatment of cell esds is used for estimating esds involving l.s. planes.

### Fractional atomic coordinates and isotropic or equivalent isotropic displacement parameters (Å^2^)

**Table:**

	*x*	*y*	*z*	*U*_iso_*/*U*_eq_	
Ti1	0.25521 (2)	0.77115 (2)	0.52922 (3)	0.01349 (10)	
N1	0.33928 (10)	0.74376 (11)	0.57924 (12)	0.0165 (4)	
N2	0.26913 (11)	0.85300 (10)	0.46239 (13)	0.0165 (4)	
C1	0.24245 (13)	0.67486 (13)	0.44812 (15)	0.0183 (5)	
C2	0.19622 (13)	0.72333 (13)	0.41991 (16)	0.0191 (5)	
C3	0.14749 (12)	0.73412 (12)	0.47990 (15)	0.0173 (5)	
C4	0.16372 (13)	0.69257 (13)	0.54563 (15)	0.0175 (5)	
C5	0.22181 (12)	0.65600 (12)	0.52647 (16)	0.0178 (5)	
C6	0.29283 (15)	0.63923 (15)	0.39780 (19)	0.0265 (6)	
H6A	0.3143	0.6711	0.3619	0.040*	
H6B	0.2705	0.6046	0.3665	0.040*	
H6C	0.3266	0.6187	0.4321	0.040*	
C7	0.19600 (15)	0.75307 (16)	0.33785 (16)	0.0257 (6)	
H7A	0.1748	0.7971	0.3395	0.039*	
H7B	0.1711	0.7239	0.3018	0.039*	
H7C	0.2420	0.7576	0.3189	0.039*	
C8	0.08424 (13)	0.77324 (14)	0.47078 (19)	0.0244 (5)	
H8A	0.0732	0.7948	0.5214	0.037*	
H8B	0.0480	0.7432	0.4554	0.037*	
H8C	0.0902	0.8073	0.4296	0.037*	
C9	0.12056 (15)	0.68160 (15)	0.61720 (18)	0.0249 (6)	
H9A	0.1486	0.6780	0.6647	0.037*	
H9B	0.0950	0.6404	0.6104	0.037*	
H9C	0.0899	0.7192	0.6233	0.037*	
C10	0.25101 (16)	0.60119 (14)	0.57619 (18)	0.0255 (6)	
H10A	0.2971	0.5929	0.5595	0.038*	
H10B	0.2246	0.5605	0.5693	0.038*	
H10C	0.2504	0.6144	0.6323	0.038*	
C11	0.38873 (12)	0.69317 (13)	0.58313 (16)	0.0160 (5)	
C12	0.40136 (14)	0.66049 (14)	0.65474 (17)	0.0212 (5)	
H12	0.3775	0.6728	0.7013	0.025*	
C13	0.44865 (15)	0.61008 (15)	0.65820 (19)	0.0255 (6)	
H13	0.4564	0.5873	0.7068	0.031*	
C14	0.48470 (14)	0.59293 (14)	0.5909 (2)	0.0259 (6)	
H14	0.5173	0.5587	0.5934	0.031*	
C15	0.47305 (14)	0.62589 (14)	0.51979 (19)	0.0236 (6)	
H15	0.4982	0.6146	0.4739	0.028*	
C16	0.42487 (13)	0.67536 (13)	0.51536 (16)	0.0196 (5)	
H16	0.4164	0.6971	0.4662	0.024*	
C17	0.34882 (13)	0.80236 (13)	0.62103 (16)	0.0184 (5)	
C18	0.41617 (13)	0.83181 (13)	0.63272 (16)	0.0167 (5)	
C19	0.46455 (13)	0.83115 (13)	0.57330 (15)	0.0188 (5)	
H19	0.4554	0.8096	0.5242	0.023*	
C20	0.52607 (14)	0.86168 (14)	0.58502 (17)	0.0216 (5)	
H20	0.5584	0.8606	0.5437	0.026*	
C21	0.54113 (14)	0.89386 (14)	0.65630 (17)	0.0214 (5)	
C22	0.49367 (14)	0.89274 (15)	0.71652 (17)	0.0227 (6)	
H22	0.5033	0.9132	0.7661	0.027*	
C23	0.43237 (14)	0.86209 (14)	0.70517 (17)	0.0207 (5)	
H23	0.4009	0.8617	0.7473	0.025*	
C24	0.60631 (16)	0.93054 (18)	0.6678 (2)	0.0332 (7)	
H24A	0.6385	0.9155	0.6278	0.050*	
H24B	0.6237	0.9213	0.7211	0.050*	
H24C	0.5989	0.9786	0.6618	0.050*	
C25	0.29109 (13)	0.83635 (13)	0.64712 (15)	0.0166 (5)	
C26	0.29111 (14)	0.90781 (13)	0.65934 (16)	0.0194 (5)	
H26	0.3302	0.9327	0.6480	0.023*	
C27	0.23609 (15)	0.93964 (14)	0.68677 (17)	0.0236 (6)	
H27	0.2377	0.9866	0.6940	0.028*	
C28	0.17566 (14)	0.90498 (16)	0.70515 (17)	0.0238 (6)	
C29	0.17200 (14)	0.83910 (16)	0.68804 (16)	0.0220 (5)	
H29	0.1320	0.8156	0.6993	0.026*	
C30	0.22712 (13)	0.80375 (14)	0.65334 (17)	0.0203 (5)	
H30	0.2255 (16)	0.7539 (16)	0.6633 (19)	0.018 (8)*	
C31	0.11923 (16)	0.94261 (18)	0.7438 (2)	0.0341 (7)	
H31A	0.1344	0.9613	0.7945	0.051*	
H31B	0.0819	0.9122	0.7534	0.051*	
H31C	0.1047	0.9788	0.7086	0.051*	
C32	0.33448 (13)	0.86856 (13)	0.43620 (16)	0.0181 (5)	
C33	0.37170 (14)	0.82304 (15)	0.39128 (15)	0.0206 (5)	
H33	0.3531	0.7811	0.3774	0.025*	
C34	0.43614 (14)	0.83944 (17)	0.36691 (17)	0.0259 (6)	
H34	0.4613	0.8082	0.3368	0.031*	
C35	0.46377 (15)	0.90029 (17)	0.38585 (19)	0.0284 (6)	
H35	0.5076	0.9112	0.3687	0.034*	
C36	0.42683 (15)	0.94556 (16)	0.4303 (2)	0.0280 (6)	
H36	0.4457	0.9875	0.4438	0.034*	
C37	0.36303 (14)	0.93018 (14)	0.45505 (18)	0.0229 (6)	
H37	0.3383	0.9617	0.4851	0.028*	
C38	0.22008 (13)	0.89928 (12)	0.43968 (15)	0.0159 (5)	
C39	0.16483 (13)	0.91041 (13)	0.48870 (16)	0.0192 (5)	
H39	0.1619	0.8885	0.5386	0.023*	
C40	0.11412 (14)	0.95335 (14)	0.46488 (19)	0.0234 (5)	
H40	0.0763	0.9595	0.4981	0.028*	
C41	0.11815 (15)	0.98732 (15)	0.39313 (18)	0.0244 (6)	
H41	0.0833	1.0164	0.3770	0.029*	
C42	0.17374 (15)	0.97823 (14)	0.34527 (17)	0.0229 (5)	
H42	0.1774	1.0023	0.2968	0.027*	
C43	0.22390 (14)	0.93457 (13)	0.36723 (16)	0.0198 (5)	
H43	0.2612	0.9283	0.3332	0.024*	

### Atomic displacement parameters (Å^2^)

**Table:**

	*U*^11^	*U*^22^	*U*^33^	*U*^12^	*U*^13^	*U*^23^
Ti1	0.0154 (2)	0.01400 (19)	0.01109 (17)	−0.00003 (14)	−0.00026 (16)	0.00082 (16)
N1	0.0164 (9)	0.0204 (10)	0.0127 (9)	0.0021 (8)	0.0002 (8)	−0.0009 (8)
N2	0.0169 (9)	0.0170 (9)	0.0156 (10)	−0.0011 (8)	−0.0004 (8)	0.0026 (8)
C1	0.0196 (12)	0.0190 (11)	0.0165 (11)	−0.0002 (9)	0.0006 (9)	−0.0032 (9)
C2	0.0232 (12)	0.0206 (12)	0.0135 (11)	−0.0013 (10)	−0.0028 (10)	−0.0017 (10)
C3	0.0177 (11)	0.0177 (11)	0.0164 (11)	−0.0027 (9)	−0.0017 (9)	−0.0027 (9)
C4	0.0192 (11)	0.0173 (11)	0.0160 (12)	−0.0052 (9)	0.0008 (9)	−0.0005 (9)
C5	0.0205 (11)	0.0149 (10)	0.0179 (11)	−0.0033 (9)	−0.0027 (10)	−0.0004 (10)
C6	0.0247 (14)	0.0273 (14)	0.0274 (15)	0.0012 (11)	0.0028 (11)	−0.0112 (12)
C7	0.0323 (15)	0.0313 (15)	0.0136 (12)	−0.0039 (12)	−0.0032 (11)	0.0005 (11)
C8	0.0197 (12)	0.0237 (12)	0.0296 (14)	0.0013 (10)	−0.0030 (12)	−0.0043 (12)
C9	0.0267 (14)	0.0287 (14)	0.0194 (13)	−0.0088 (11)	0.0063 (11)	−0.0003 (11)
C10	0.0305 (14)	0.0181 (12)	0.0279 (14)	−0.0017 (11)	−0.0062 (12)	0.0037 (11)
C11	0.0151 (11)	0.0166 (11)	0.0161 (11)	−0.0006 (9)	−0.0016 (9)	−0.0023 (9)
C12	0.0219 (13)	0.0221 (13)	0.0196 (12)	0.0003 (10)	−0.0024 (10)	0.0001 (10)
C13	0.0254 (14)	0.0212 (13)	0.0300 (15)	0.0004 (11)	−0.0085 (12)	0.0039 (11)
C14	0.0198 (13)	0.0180 (12)	0.0399 (17)	0.0018 (10)	−0.0038 (12)	−0.0043 (12)
C15	0.0212 (12)	0.0221 (12)	0.0276 (15)	−0.0021 (10)	0.0033 (11)	−0.0081 (11)
C16	0.0204 (12)	0.0218 (12)	0.0167 (13)	0.0004 (9)	0.0004 (10)	−0.0040 (10)
C17	0.0166 (11)	0.0199 (12)	0.0188 (12)	−0.0003 (9)	−0.0004 (10)	−0.0005 (10)
C18	0.0168 (11)	0.0162 (11)	0.0170 (12)	0.0001 (9)	0.0013 (9)	0.0000 (9)
C19	0.0219 (12)	0.0215 (12)	0.0131 (11)	0.0005 (10)	0.0007 (10)	−0.0008 (10)
C20	0.0206 (12)	0.0253 (13)	0.0188 (12)	−0.0009 (10)	0.0056 (10)	−0.0014 (11)
C21	0.0190 (12)	0.0220 (12)	0.0231 (13)	−0.0008 (10)	0.0026 (11)	−0.0042 (11)
C22	0.0227 (13)	0.0279 (14)	0.0176 (12)	−0.0010 (11)	0.0009 (11)	−0.0067 (11)
C23	0.0206 (13)	0.0238 (13)	0.0175 (13)	0.0003 (10)	0.0026 (10)	−0.0028 (10)
C24	0.0207 (14)	0.0389 (18)	0.0401 (19)	−0.0081 (12)	0.0040 (13)	−0.0120 (15)
C25	0.0182 (11)	0.0198 (12)	0.0120 (11)	−0.0008 (9)	0.0000 (9)	0.0007 (9)
C26	0.0208 (12)	0.0193 (12)	0.0179 (12)	−0.0009 (9)	−0.0010 (10)	0.0001 (10)
C27	0.0260 (14)	0.0214 (12)	0.0235 (13)	0.0049 (11)	−0.0050 (11)	−0.0060 (10)
C28	0.0216 (13)	0.0356 (16)	0.0142 (12)	0.0077 (11)	−0.0013 (10)	−0.0070 (11)
C29	0.0173 (12)	0.0330 (15)	0.0156 (12)	0.0003 (10)	0.0015 (10)	−0.0011 (11)
C30	0.0185 (11)	0.0215 (12)	0.0210 (13)	−0.0004 (10)	0.0003 (10)	0.0007 (10)
C31	0.0248 (15)	0.0434 (19)	0.0342 (17)	0.0030 (13)	0.0025 (13)	−0.0174 (15)
C32	0.0177 (12)	0.0214 (12)	0.0154 (12)	0.0002 (9)	−0.0008 (9)	0.0051 (10)
C33	0.0231 (13)	0.0270 (13)	0.0119 (11)	0.0014 (10)	−0.0018 (10)	0.0019 (10)
C34	0.0209 (13)	0.0418 (17)	0.0150 (12)	0.0050 (12)	0.0000 (10)	0.0056 (12)
C35	0.0194 (13)	0.0415 (17)	0.0243 (14)	−0.0005 (12)	0.0009 (11)	0.0130 (13)
C36	0.0224 (13)	0.0272 (15)	0.0345 (17)	−0.0062 (11)	−0.0035 (12)	0.0108 (13)
C37	0.0208 (12)	0.0207 (12)	0.0272 (15)	0.0009 (10)	−0.0002 (11)	0.0029 (11)
C38	0.0180 (11)	0.0143 (10)	0.0154 (11)	−0.0016 (9)	−0.0013 (9)	0.0003 (9)
C39	0.0205 (12)	0.0179 (12)	0.0192 (13)	−0.0010 (9)	0.0029 (10)	0.0034 (9)
C40	0.0212 (12)	0.0230 (12)	0.0259 (14)	0.0009 (10)	0.0029 (11)	0.0020 (12)
C41	0.0234 (13)	0.0237 (13)	0.0262 (14)	0.0048 (11)	−0.0050 (11)	0.0014 (11)
C42	0.0302 (14)	0.0221 (13)	0.0164 (12)	0.0018 (11)	−0.0020 (11)	0.0018 (10)
C43	0.0239 (13)	0.0203 (12)	0.0152 (12)	0.0008 (10)	0.0005 (10)	0.0008 (9)

### Geometric parameters (Å, º)

**Table:**

Ti1—N1	1.963 (2)	C17—C18	1.488 (4)
Ti1—N2	2.009 (2)	C18—C19	1.393 (4)
Ti1—C30	2.259 (3)	C18—C23	1.400 (4)
Ti1—C1	2.379 (3)	C19—C20	1.392 (4)
Ti1—C2	2.387 (3)	C19—H19	0.9500
Ti1—C5	2.406 (2)	C20—C21	1.394 (4)
Ti1—C3	2.431 (2)	C20—H20	0.9500
Ti1—C4	2.435 (3)	C21—C22	1.390 (4)
Ti1—C25	2.482 (3)	C21—C24	1.513 (4)
Ti1—C17	2.511 (3)	C22—C23	1.388 (4)
N1—C17	1.383 (3)	C22—H22	0.9500
N1—C11	1.421 (3)	C23—H23	0.9500
N2—C38	1.406 (3)	C24—H24A	0.9800
N2—C32	1.418 (3)	C24—H24B	0.9800
C1—C2	1.425 (4)	C24—H24C	0.9800
C1—C5	1.432 (4)	C25—C30	1.444 (4)
C1—C6	1.500 (4)	C25—C26	1.448 (4)
C2—C3	1.421 (4)	C26—C27	1.356 (4)
C2—C7	1.503 (4)	C26—H26	0.9500
C3—C4	1.422 (4)	C27—C28	1.432 (4)
C3—C8	1.500 (4)	C27—H27	0.9500
C4—C5	1.414 (4)	C28—C29	1.355 (4)
C4—C9	1.499 (4)	C28—C31	1.508 (4)
C5—C10	1.501 (4)	C29—C30	1.438 (4)
C6—H6A	0.9800	C29—H29	0.9500
C6—H6B	0.9800	C30—H30	1.01 (3)
C6—H6C	0.9800	C31—H31A	0.9800
C7—H7A	0.9800	C31—H31B	0.9800
C7—H7B	0.9800	C31—H31C	0.9800
C7—H7C	0.9800	C32—C37	1.399 (4)
C8—H8A	0.9800	C32—C33	1.401 (4)
C8—H8B	0.9800	C33—C34	1.396 (4)
C8—H8C	0.9800	C33—H33	0.9500
C9—H9A	0.9800	C34—C35	1.378 (5)
C9—H9B	0.9800	C34—H34	0.9500
C9—H9C	0.9800	C35—C36	1.390 (5)
C10—H10A	0.9800	C35—H35	0.9500
C10—H10B	0.9800	C36—C37	1.381 (4)
C10—H10C	0.9800	C36—H36	0.9500
C11—C12	1.394 (4)	C37—H37	0.9500
C11—C16	1.397 (4)	C38—C39	1.399 (4)
C12—C13	1.388 (4)	C38—C43	1.411 (4)
C12—H12	0.9500	C39—C40	1.392 (4)
C13—C14	1.387 (5)	C39—H39	0.9500
C13—H13	0.9500	C40—C41	1.388 (4)
C14—C15	1.386 (5)	C40—H40	0.9500
C14—H14	0.9500	C41—C42	1.387 (4)
C15—C16	1.387 (4)	C41—H41	0.9500
C15—H15	0.9500	C42—C43	1.384 (4)
C16—H16	0.9500	C42—H42	0.9500
C17—C25	1.414 (4)	C43—H43	0.9500
			
N1—Ti1—N2	110.42 (9)	C16—C11—N1	120.5 (2)
N1—Ti1—C30	84.23 (9)	C13—C12—C11	120.2 (3)
N2—Ti1—C30	108.35 (9)	C13—C12—H12	119.9
N1—Ti1—C1	96.36 (9)	C11—C12—H12	119.9
N2—Ti1—C1	110.98 (9)	C14—C13—C12	120.2 (3)
C30—Ti1—C1	137.58 (10)	C14—C13—H13	119.9
N1—Ti1—C2	130.03 (9)	C12—C13—H13	119.9
N2—Ti1—C2	88.09 (9)	C15—C14—C13	119.9 (3)
C30—Ti1—C2	134.76 (10)	C15—C14—H14	120.1
C1—Ti1—C2	34.80 (9)	C13—C14—H14	120.1
N1—Ti1—C5	88.78 (9)	C14—C15—C16	120.3 (3)
N2—Ti1—C5	144.36 (9)	C14—C15—H15	119.8
C30—Ti1—C5	103.08 (10)	C16—C15—H15	119.8
C1—Ti1—C5	34.82 (9)	C15—C16—C11	120.0 (3)
C2—Ti1—C5	57.40 (9)	C15—C16—H16	120.0
N1—Ti1—C3	145.52 (9)	C11—C16—H16	120.0
N2—Ti1—C3	100.51 (9)	N1—C17—C25	117.0 (2)
C30—Ti1—C3	100.49 (9)	N1—C17—C18	122.0 (2)
C1—Ti1—C3	57.35 (9)	C25—C17—C18	120.8 (2)
C2—Ti1—C3	34.29 (9)	N1—C17—Ti1	51.10 (12)
C5—Ti1—C3	56.80 (9)	C25—C17—Ti1	72.43 (15)
N1—Ti1—C4	114.69 (9)	C18—C17—Ti1	149.29 (19)
N2—Ti1—C4	134.20 (9)	C19—C18—C23	117.8 (2)
C30—Ti1—C4	83.96 (9)	C19—C18—C17	122.3 (2)
C1—Ti1—C4	57.18 (9)	C23—C18—C17	119.9 (2)
C2—Ti1—C4	56.87 (9)	C20—C19—C18	120.8 (2)
C5—Ti1—C4	33.96 (8)	C20—C19—H19	119.6
C3—Ti1—C4	33.99 (9)	C18—C19—H19	119.6
N1—Ti1—C25	63.65 (9)	C19—C20—C21	121.2 (2)
N2—Ti1—C25	88.61 (9)	C19—C20—H20	119.4
C30—Ti1—C25	35.07 (9)	C21—C20—H20	119.4
C1—Ti1—C25	156.53 (9)	C22—C21—C20	118.1 (3)
C2—Ti1—C25	166.07 (9)	C22—C21—C24	120.5 (3)
C5—Ti1—C25	127.03 (9)	C20—C21—C24	121.5 (3)
C3—Ti1—C25	133.74 (9)	C23—C22—C21	120.9 (3)
C4—Ti1—C25	117.99 (8)	C23—C22—H22	119.5
N1—Ti1—C17	33.24 (9)	C21—C22—H22	119.5
N2—Ti1—C17	92.07 (9)	C22—C23—C18	121.2 (3)
C30—Ti1—C17	63.01 (9)	C22—C23—H23	119.4
C1—Ti1—C17	129.47 (9)	C18—C23—H23	119.4
C2—Ti1—C17	160.82 (9)	C21—C24—H24A	109.5
C5—Ti1—C17	117.37 (9)	C21—C24—H24B	109.5
C3—Ti1—C17	162.00 (9)	H24A—C24—H24B	109.5
C4—Ti1—C17	130.97 (9)	C21—C24—H24C	109.5
C25—Ti1—C17	32.90 (8)	H24A—C24—H24C	109.5
C17—N1—C11	119.2 (2)	H24B—C24—H24C	109.5
C17—N1—Ti1	95.66 (16)	C17—C25—C30	122.1 (2)
C11—N1—Ti1	145.04 (18)	C17—C25—C26	121.4 (2)
C38—N2—C32	114.7 (2)	C30—C25—C26	116.0 (2)
C38—N2—Ti1	126.47 (17)	C17—C25—Ti1	74.67 (15)
C32—N2—Ti1	118.83 (16)	C30—C25—Ti1	63.98 (14)
C2—C1—C5	107.4 (2)	C26—C25—Ti1	129.43 (18)
C2—C1—C6	125.2 (2)	C27—C26—C25	120.9 (3)
C5—C1—C6	126.0 (2)	C27—C26—H26	119.5
C2—C1—Ti1	72.94 (15)	C25—C26—H26	119.5
C5—C1—Ti1	73.64 (14)	C26—C27—C28	122.3 (3)
C6—C1—Ti1	129.66 (19)	C26—C27—H27	118.9
C3—C2—C1	108.4 (2)	C28—C27—H27	118.9
C3—C2—C7	126.1 (2)	C29—C28—C27	118.3 (3)
C1—C2—C7	125.3 (3)	C29—C28—C31	122.6 (3)
C3—C2—Ti1	74.53 (15)	C27—C28—C31	119.1 (3)
C1—C2—Ti1	72.26 (15)	C28—C29—C30	121.7 (3)
C7—C2—Ti1	123.27 (19)	C28—C29—H29	119.1
C2—C3—C4	107.7 (2)	C30—C29—H29	119.1
C2—C3—C8	126.1 (2)	C29—C30—C25	119.3 (2)
C4—C3—C8	125.4 (2)	C29—C30—Ti1	135.2 (2)
C2—C3—Ti1	71.17 (14)	C25—C30—Ti1	80.95 (16)
C4—C3—Ti1	73.17 (14)	C29—C30—H30	113.2 (19)
C8—C3—Ti1	128.83 (18)	C25—C30—H30	119.2 (19)
C5—C4—C3	108.4 (2)	Ti1—C30—H30	82.8 (19)
C5—C4—C9	125.6 (2)	C28—C31—H31A	109.5
C3—C4—C9	125.3 (2)	C28—C31—H31B	109.5
C5—C4—Ti1	71.88 (14)	H31A—C31—H31B	109.5
C3—C4—Ti1	72.85 (14)	C28—C31—H31C	109.5
C9—C4—Ti1	128.42 (18)	H31A—C31—H31C	109.5
C4—C5—C1	108.1 (2)	H31B—C31—H31C	109.5
C4—C5—C10	125.1 (3)	C37—C32—C33	118.7 (3)
C1—C5—C10	126.4 (2)	C37—C32—N2	120.2 (2)
C4—C5—Ti1	74.15 (14)	C33—C32—N2	121.1 (2)
C1—C5—Ti1	71.54 (14)	C34—C33—C32	119.9 (3)
C10—C5—Ti1	125.75 (18)	C34—C33—H33	120.1
C1—C6—H6A	109.5	C32—C33—H33	120.1
C1—C6—H6B	109.5	C35—C34—C33	120.9 (3)
H6A—C6—H6B	109.5	C35—C34—H34	119.5
C1—C6—H6C	109.5	C33—C34—H34	119.5
H6A—C6—H6C	109.5	C34—C35—C36	119.2 (3)
H6B—C6—H6C	109.5	C34—C35—H35	120.4
C2—C7—H7A	109.5	C36—C35—H35	120.4
C2—C7—H7B	109.5	C37—C36—C35	120.7 (3)
H7A—C7—H7B	109.5	C37—C36—H36	119.7
C2—C7—H7C	109.5	C35—C36—H36	119.7
H7A—C7—H7C	109.5	C36—C37—C32	120.6 (3)
H7B—C7—H7C	109.5	C36—C37—H37	119.7
C3—C8—H8A	109.5	C32—C37—H37	119.7
C3—C8—H8B	109.5	C39—C38—N2	120.0 (2)
H8A—C8—H8B	109.5	C39—C38—C43	118.1 (2)
C3—C8—H8C	109.5	N2—C38—C43	121.9 (2)
H8A—C8—H8C	109.5	C40—C39—C38	120.5 (2)
H8B—C8—H8C	109.5	C40—C39—H39	119.7
C4—C9—H9A	109.5	C38—C39—H39	119.7
C4—C9—H9B	109.5	C41—C40—C39	120.8 (3)
H9A—C9—H9B	109.5	C41—C40—H40	119.6
C4—C9—H9C	109.5	C39—C40—H40	119.6
H9A—C9—H9C	109.5	C42—C41—C40	119.1 (3)
H9B—C9—H9C	109.5	C42—C41—H41	120.4
C5—C10—H10A	109.5	C40—C41—H41	120.4
C5—C10—H10B	109.5	C43—C42—C41	120.9 (3)
H10A—C10—H10B	109.5	C43—C42—H42	119.6
C5—C10—H10C	109.5	C41—C42—H42	119.6
H10A—C10—H10C	109.5	C42—C43—C38	120.6 (3)
H10B—C10—H10C	109.5	C42—C43—H43	119.7
C12—C11—C16	119.3 (2)	C38—C43—H43	119.7
C12—C11—N1	120.2 (2)		
			
C5—C1—C2—C3	−0.2 (3)	N1—C17—C18—C23	−142.0 (3)
C6—C1—C2—C3	166.8 (3)	C25—C17—C18—C23	43.1 (4)
Ti1—C1—C2—C3	−66.34 (18)	Ti1—C17—C18—C23	151.4 (3)
C5—C1—C2—C7	−175.2 (2)	C23—C18—C19—C20	−2.1 (4)
C6—C1—C2—C7	−8.3 (4)	C17—C18—C19—C20	177.2 (3)
Ti1—C1—C2—C7	118.6 (3)	C18—C19—C20—C21	−0.1 (4)
C5—C1—C2—Ti1	66.13 (17)	C19—C20—C21—C22	2.1 (4)
C6—C1—C2—Ti1	−126.9 (3)	C19—C20—C21—C24	−176.4 (3)
C1—C2—C3—C4	0.3 (3)	C20—C21—C22—C23	−1.8 (4)
C7—C2—C3—C4	175.3 (2)	C24—C21—C22—C23	176.7 (3)
Ti1—C2—C3—C4	−64.51 (17)	C21—C22—C23—C18	−0.5 (4)
C1—C2—C3—C8	−170.3 (2)	C19—C18—C23—C22	2.4 (4)
C7—C2—C3—C8	4.7 (4)	C17—C18—C23—C22	−176.9 (3)
Ti1—C2—C3—C8	124.8 (3)	N1—C17—C25—C30	19.1 (4)
C1—C2—C3—Ti1	64.84 (18)	C18—C17—C25—C30	−165.8 (2)
C7—C2—C3—Ti1	−120.2 (3)	Ti1—C17—C25—C30	44.8 (2)
C2—C3—C4—C5	−0.3 (3)	N1—C17—C25—C26	−152.9 (2)
C8—C3—C4—C5	170.4 (2)	C18—C17—C25—C26	22.3 (4)
Ti1—C3—C4—C5	−63.53 (17)	Ti1—C17—C25—C26	−127.2 (2)
C2—C3—C4—C9	−171.5 (2)	N1—C17—C25—Ti1	−25.7 (2)
C8—C3—C4—C9	−0.7 (4)	C18—C17—C25—Ti1	149.4 (2)
Ti1—C3—C4—C9	125.3 (3)	C17—C25—C26—C27	−177.9 (3)
C2—C3—C4—Ti1	63.20 (18)	C30—C25—C26—C27	9.6 (4)
C8—C3—C4—Ti1	−126.1 (2)	Ti1—C25—C26—C27	86.3 (3)
C3—C4—C5—C1	0.2 (3)	C25—C26—C27—C28	0.1 (4)
C9—C4—C5—C1	171.3 (2)	C26—C27—C28—C29	−5.7 (4)
Ti1—C4—C5—C1	−63.95 (17)	C26—C27—C28—C31	173.3 (3)
C3—C4—C5—C10	−173.0 (2)	C27—C28—C29—C30	0.9 (4)
C9—C4—C5—C10	−1.9 (4)	C31—C28—C29—C30	−178.1 (3)
Ti1—C4—C5—C10	122.9 (3)	C28—C29—C30—C25	9.2 (4)
C3—C4—C5—Ti1	64.16 (17)	C28—C29—C30—Ti1	−98.8 (3)
C9—C4—C5—Ti1	−124.8 (3)	C17—C25—C30—C29	173.6 (2)
C2—C1—C5—C4	0.0 (3)	C26—C25—C30—C29	−14.1 (4)
C6—C1—C5—C4	−166.8 (3)	Ti1—C25—C30—C29	−137.3 (3)
Ti1—C1—C5—C4	65.66 (17)	C17—C25—C30—Ti1	−49.1 (2)
C2—C1—C5—C10	173.1 (2)	C26—C25—C30—Ti1	123.2 (2)
C6—C1—C5—C10	6.2 (4)	C38—N2—C32—C37	55.4 (3)
Ti1—C1—C5—C10	−121.3 (3)	Ti1—N2—C32—C37	−122.8 (2)
C2—C1—C5—Ti1	−65.66 (18)	C38—N2—C32—C33	−124.6 (3)
C6—C1—C5—Ti1	127.5 (3)	Ti1—N2—C32—C33	57.2 (3)
C17—N1—C11—C12	65.8 (3)	C37—C32—C33—C34	0.6 (4)
Ti1—N1—C11—C12	−119.8 (3)	N2—C32—C33—C34	−179.4 (2)
C17—N1—C11—C16	−114.5 (3)	C32—C33—C34—C35	−0.5 (4)
Ti1—N1—C11—C16	59.9 (4)	C33—C34—C35—C36	0.4 (4)
C16—C11—C12—C13	−1.0 (4)	C34—C35—C36—C37	−0.3 (5)
N1—C11—C12—C13	178.6 (2)	C35—C36—C37—C32	0.3 (5)
C11—C12—C13—C14	1.5 (4)	C33—C32—C37—C36	−0.5 (4)
C12—C13—C14—C15	−0.5 (4)	N2—C32—C37—C36	179.5 (3)
C13—C14—C15—C16	−0.9 (4)	C32—N2—C38—C39	−148.6 (2)
C14—C15—C16—C11	1.3 (4)	Ti1—N2—C38—C39	29.5 (3)
C12—C11—C16—C15	−0.4 (4)	C32—N2—C38—C43	32.7 (3)
N1—C11—C16—C15	180.0 (2)	Ti1—N2—C38—C43	−149.2 (2)
C11—N1—C17—C25	−151.1 (2)	N2—C38—C39—C40	−176.5 (2)
Ti1—N1—C17—C25	32.1 (2)	C43—C38—C39—C40	2.3 (4)
C11—N1—C17—C18	33.8 (4)	C38—C39—C40—C41	−1.7 (4)
Ti1—N1—C17—C18	−143.0 (2)	C39—C40—C41—C42	−0.3 (4)
C11—N1—C17—Ti1	176.8 (3)	C40—C41—C42—C43	1.8 (4)
N1—C17—C18—C19	38.7 (4)	C41—C42—C43—C38	−1.2 (4)
C25—C17—C18—C19	−136.2 (3)	C39—C38—C43—C42	−0.8 (4)
Ti1—C17—C18—C19	−27.9 (5)	N2—C38—C43—C42	177.9 (2)
